# Fibroblast-like cells change gene expression of bone remodelling markers in transwell cultures

**DOI:** 10.1186/s40001-020-00453-y

**Published:** 2020-10-29

**Authors:** Eliza S. Hartmann, Sabine Schluessel, Miriam I. Köhler, Felicitas Beck, Julia I. Redeker, Burkhard Summer, Veronika Schönitzer, Andreas Fottner, Susanne Mayer-Wagner

**Affiliations:** 1 Department of Orthopaedics, Physical Medicine and Rehabilitation, University Hospital, LMU Munich, Marchioninistr. 15, 81377 Munich, Germany; 2 Department of Dermatology, Ludwig-Maximilians-University, Frauenlobstr. 9-11, 80337 Munich, Germany; 3 Department of Surgery, Experimental Surgery, and Regenerative Medicine, Ludwig-Maximilians-University, Nußbaumstr. 20, 80337 Munich, Germany

**Keywords:** Implant material, Fibroblast-like cells, Transwell culture, Bone remodeling, Aseptic loosening

## Abstract

**Introduction:**

Periprosthetic fibroblast-like cells (PPFs) play an important role in aseptic loosening of arthroplasties. Various studies have examined PPF behavior in monolayer culture systems. However, the periprosthetic tissue is a three-dimensional (3D) mesh, which allows the cells to interact in a multidirectional way. The expression of bone remodeling markers of fibroblast-like cells in a multilayer environment changes significantly versus monolayer cultures without the addition of particles or cytokine stimulation. Gene expression of bone remodeling markers was therefore compared in fibroblast-like cells from different origins and dermal fibroblasts under transwell culture conditions versus monolayer cultures.

**Methods:**

PPFs from periprosthetic tissues (*n* = 12), osteoarthritic (OA) synovial fibroblast-like cells (SFs) (*n* = 6), and dermal fibroblasts (DFs) were cultured in monolayer (density 5.5 × 10^3^/cm^2^) or multilayer cultures (density 8.5 × 10^5^/cm^2^) for 10 or 21 days. Cultures were examined via histology, TRAP staining, immunohistochemistry (anti-S100a4), and quantitative real-time PCR.

**Results:**

Fibroblast-like cells (PPFs/SFs) and dermal fibroblasts significantly increased the expression of RANKL and significantly decreased the expression of ALP, COL1A1, and OPG in multilayer cultures. PPFs and SFs in multilayer cultures further showed a higher expression of cathepsin K, MMP-13, and TNF-α. In multilayer PPF cultures, the mRNA level of TRAP was also found to be significantly increased.

**Conclusion:**

The multilayer cultures are able to induce significant expression changes in fibroblast-like cells depending on the nature of cellular origin without the addition of any further stimulus. This system might be a useful tool to get more in vivo like results regarding fibroblast-like cell cultures.

## Background

Various materials are used in implant applications. However, the fail of implants always leads to the common process of periprosthetic osteolysis, which is a multifactorial process [1–3]. Osteoclastogenesis is induced due to an inflammatory response to miscellaneous implant particles. A pseudomembrane forms, which consists of various cellular compounds predominantly of fibroblasts, osteoclasts, giant cells, inflammatory cells, and macrophages [4]. Although osteoclasts claim the leading role in periprosthetic osteolysis, other cellular compounds have been shown to be involved [5, 6]. The periprosthetic tissue is lined by a synovium-like interface membrane, which consists of synovial-like lining cells. The synovial-like lining cells form the surface of the synovium-like interface, which consists mainly of periprosthetic fibroblast-like cells (PPFs). The PPFs have been shown to play an active role in periprosthetic osteolysis [7].

PPFs have been shown to play an active role in periprosthetic osteolysis [7], which is astonishing regarding their fibroblastic nature. PPFs are able to phagocytose many types of particles both in vivo and in vitro [4]. PPFs have been demonstrated to change the molecular surrounding by expressing under certain conditions cathepsin K [8], matrix metalloproteinase 13 (MMP-13) [9], NF‐κB‐Ligand (RANKL) [4, 6, 8, 10], macrophage colony-stimulating factor (M-CSF) [4], osteoprotegerin (OPG) [4, 8], and tumor necrosis factor‐α (TNF-α) [11]. Although fibroblast-like cells are less effective than osteoblasts, PPFs seem to have the capacity to induce osteoclastogenesis [10]. Fibroblast-like cells of various origins have been considered as therapeutic targets in the treatment of bone destructive diseases [12, 13]. In periprosthetic tissue, fibroblast-like cells form a complex fibrous 3D mesh in which the cells are usually surrounded by extracellular matrix, bone, and endoprosthetic compounds. Transwell cultures are well known to investigate cellular interactions and have been established for fibroblast multilayer cultures [14].

Culturing fibroblasts within a transwell system may lead to higher cell mass and increased cell numbers [15]. The conglomerates formed within transwell cultures are partial three-dimensional and multilayered, increasing the intercellular cell contacts within the cellular structures. The matrix elasticity of the membrane further stimulates the differentiation [16]. We showed that PPFs co-cultured with PBMCs in transwell cultures resulted in a considerably more osteoclastogenic gene expression profile, being closer to in vivo findings of periosteolytic tissues [17].

However, most in vitro studies of PPF and other fibroblast-like cell behavior have been performed using monolayer cultures, which lack the multilayer component of the original tissue. To analyze cells in a more natural cellular environment, multilayer cultures using an optimal medium supply from both sides have been implemented which enforce intercellular connections and direct cell–cell contact. In this study, the molecular biology and expression of bone remodeling markers of fibroblast-like cells in multilayer cultures was analyzed without the addition of a further stimulus.

## Materials and methods

### Patients

Periprosthetic tissues (PPT) from 12 patients (6 male, 6 female; mean age 68; range 45–86) undergoing revision surgery of knee (total knee arthroplasty (TKA), *n* = 5) or hip (total hip arthroplasty (THA), *n* = 7) endoprostheses due to aseptic loosening were collected. Implantation techniques were various as it was the main aim to detect new pathways in aseptic loosening of arthroplasties [18]. Synovial tissues (ST) were collected from 6 patients (3 male, 3 female; mean age 61; range 45–77) undergoing hip replacement due to osteoarthritis (OA). Patients with autoimmune diseases, rheumatoid arthritis, or other inflammatory arthritis were excluded on the basis of patient history, radiological, and laboratory data. Septical loosening was excluded by microbiological cultures, which were performed at the Department of Microbiology of the Ludwig-Maximilians-University of Munich. Tissue samples were immediately incubated in the operating room in Dulbecco´s Modified eagle medium (DMEM; Biochrom, Berlin, Germany) with 60 IU/ml penicillin, 60 µg/ml streptomycin (Biochrom), and 0.25 µg/ml Amphotericin B (Sigma–Aldrich Co., St. Louis, MO).

### Isolation of fibroblast-like cells

Tissue was collected from patients as described above, washed several times with phosphate-buffered saline (PBS; Biochrom), cut into small pieces, and digested with α-Minimal Essential Medium (α-MEM; Biochrom) containing 1 mg/ml collagenase (Sigma-Aldrich Co.) for 30 min at 37 °C. Another digestion step of tissue followed for 1 h at 37 °C with Versene^®^ (Invitrogen, Darmstadt, Germany). The completely digested tissue was then filtered through a 70 µm cell strainer (BD Bioscience, San Jose, USA). Then, the digested tissue was centrifuged (1500 rcf, 10 min). The pellet was resuspended in culture medium (α-MEM) supplemented with 60 IU/ml penicillin, 60 µg/ml streptomycin, 2 mM l-Glutamin (Biochrom), and 10% foetal bovine serum (FBS; PAA Laboratories, Cölbe, Germany), and cultured in a T75 culture flask (Nunc, Roskilde, Denmark) at 37 °C and 5% CO_2_. The medium was changed twice a week. Cells were passaged at 80–90% confluence by using 0.05% trypsin (Biochrom) containing 0.02% ethylenediaminetetraacetic acid (Biochrom). Afterwards, cells were used in passage four for the following monolayer and transwell culture experiments. Cultures were also assessed histochemically for the presence of tartrate-resistant acid phosphatase (TRAP) using a TRAP detection kit (Sigma-Aldrich Co.) to exclude the presence of TRAP-positive cells.

### Monolayer culture and transwell culture of fibroblast-like cells

Fibroblast-like cells (4 × 10^5^) were seeded in a T75 culture flash (density 5.5 × 10^3^/cm^2^) for monolayer cultures. For multilayer transwell cultures, 4 × 10^5^ fibroblast-like cells were pipetted onto a polycarbonate membrane (density 8.5 × 10^5^/cm^2^) with 0.4 µm pore size (to inhibit cellular migration), spun for 5 min with 600 rcf and cultured in a transwell plate (all Nunc). Dermal human fibroblasts (HFIB-D; Provitro, Berlin, Germany) were used as control (*n* = 2). Experiments were performed in 6 groups: (1) monolayer SFs; (2) transwell SFs; (3) monolayer PPFs; (4) transwell PPFs; (5) monolayer DFs; (6) transwell DFs. They were all cultured for 10 and 21 days in culture medium and the medium change was carried out at 2-day intervals.

### Immunohistochemistry

For labeling fibroblast-like cells, the polyclonal antibody antiS100a4 ab27957 (Abcam, Cambridge, UK) was used.

Transwell cultures were embedded after 10 and 21 days in Tissue-Tek (Sakura, Zoeterwoude, The Netherlands) and frozen at − 20 °C. Samples were cut into 10 µm serial-sections, mounted on superfrost glass slides (Menzel, Braunschweig, Germany), fixed in acetone, and dried at room temperature. After re-hydrating with 0.2% Tween (Merck, Darmstadt, Germany) in PBS sections were incubated for 1 h at RT with ABO-Serum (Biotest, Dreieich, Germany) mixed with horse serum (both 1:20 in PBS with 3% Bovine Serum Albumin (BSA; Sigma-Aldrich Co.) to block unspecific binding sites. Incubation with the first antibody (AB) anti-S100A4 (Abcam) (1:100 in AB dilution buffer (DCS Innovative, Hamburg, Germany)) for 1 h at RT was performed. After washing, incubation with the biotinylated goat anti-rabbit AB (Vector Laboratories, Burlingame, USA) (1:200 dilution with AB dilution solution) was performed for 30 min at RT. After washing, the sections were treated with avidin–biotin–peroxidase complex (ABC; Vector Laboratories) for 30 min at RT. Then, the sections were washed. Staining was developed using 3-Amino-9-Ethyl Carbazol (Sigma-Aldrich Co.) and counterstained with hemalaun. The sections were then embedded in Aquamount (Merck, Darmstadt, Germany).

### Quantitative real-time PCR (qRT-PCR)

For RNA isolation, transwell cultures were disrupted under frozen conditions at 3000 rpm by a Micro-Dismembrator S (Sartorius, Göttingen, Germany). In monolayer cultures cells were lysed with 2-mercaptoethanol (Sigma-Aldrich Co.). For transwell and monolayer cultures, RNeasy Mini Kit (Qiagen, Venlo, The Netherlands) was used for RNA isolation. 0.1–0.5 µg RNA was synthesized with the QuantiTect^®^ reverse transcription kit (Qiagen). For qRT-PCR, a Light Cycler^®^ (Roche, Indianapolis, USA) was used. Gene expression analysis of the following markers was performed: M-CSF [19], TNF-α [19], RANKL [19], OPG [19], cathepsin K [20], MMP-13 [21], ALP [22], collagen type 1 (COL1A1) [19], and glyceraldehyde 3-phosphate dehydrogenase (GAPDH) were used as housekeeping gene [23]. Sequences are shown in Table 1. The amplification reactions were performed with the Light cycler^®^ Fast Start Essential DNA Master Kit (Roche). Time, temperature, and concentration of each primer are shown in Table 1. For qRT-PCR, 2.5 µL of diluted samples were used. Reactions were performed in triplicates. For the relative quantification, the delta–delta CP (2^ΔΔCP^) method was used, with day 0 as the reference sample [24].

**Table 1:** Primer, sequences, and protocols for quantitative real-time polymerase chain reaction

Primer	Sequenzen (5′-3′)	Primer concentration (nM)	Amplification	Efficiency (*E* = 10^−1/slope^)
ALP	TCAAGGGTCAGCTCCACCACA ATTGGCCTTCACCCCACACA	300	95 °C 10 s, 60 °C 10 s, 72 °C 30 s for 45 cycles	2.01
Collagen 1A1	GCTTCCCTGGTCTTCCTG TCTCACCACGGTCACCCT	500	95 °C 10 s, 65 °C 10 s, 72 °C 15 s for 40 cycles	2.01
GAPDH	TGCACCACCAACTGCTTAGC GGCATGGACTGTGGTCATGAG	300	95 °C 10 s, 60 °C 10 s, 72 °C 15 s for 40 cycles	2.04
Cathepsin K	TTCCCGCAGTAATGACACC TTTCCCCAGTTTTCTCCCC	500	95 °C 10 s, 63 °C 10 s, 72 °C 20 s for 40 cycles	1.83
M-CSF	CCGAGGAGGTGTCGGAGTAC AATTTGGCACGAGGTCTCCAT	300	95 °C 10 s, 60 °C 10 s, 72 °C 15 s for 40 cycles	2.01
MMP 13	GACTTCACGATGGCATTGCTG GCATCAACCTGCTGAGGATGC	300	95 °C 10 s, 62 °C 10 s, 72 °C 20 s for 40 cycles	1.90
OPG	CTGCGCGCTCGTGTTTC ACAGCTGATGAGAGGTTTCTTCGT	300	95 °C 30 s, 60 °C 60 s, 72 °C 15 s for 40 cycles	1.99
RANK-L	CATCCCATCTGGTTCCCATAA GCCCAACCCCGATCATG	300	95 °C 10 s, 60 °C 10 s, 72 °C 15 s for 40 cycles	2.07
TNF α	CCCAGGGACCTCTCTCTAATC GCTTGAGGGTTTGCTACAACATG	300	95 °C 30 s, 60 °C 60 s, 72 °C 15 s for 40 cycles	1.97

### Statistical analysis

Graph Pad Prism 5.01 (GraphPad Software, La Jolla, CA, USA) was utilized to analyze the data. The non-parametric Mann–Whitney U test was used to determine significant differences between the cohorts. For time-dependent analysis of PPFs and SFs, the Wilcoxon signed-rank test was used. *p* value < 0.05 was considered significant.

### Ethical approval and consent to participate

The study was approved by the responsible Ludwig-Maximilians-University medical center ethics committee. Based on the study design using disposable material, no patient consent was required.

## Results

### Immunohistochemistry

**Fig. 1:**
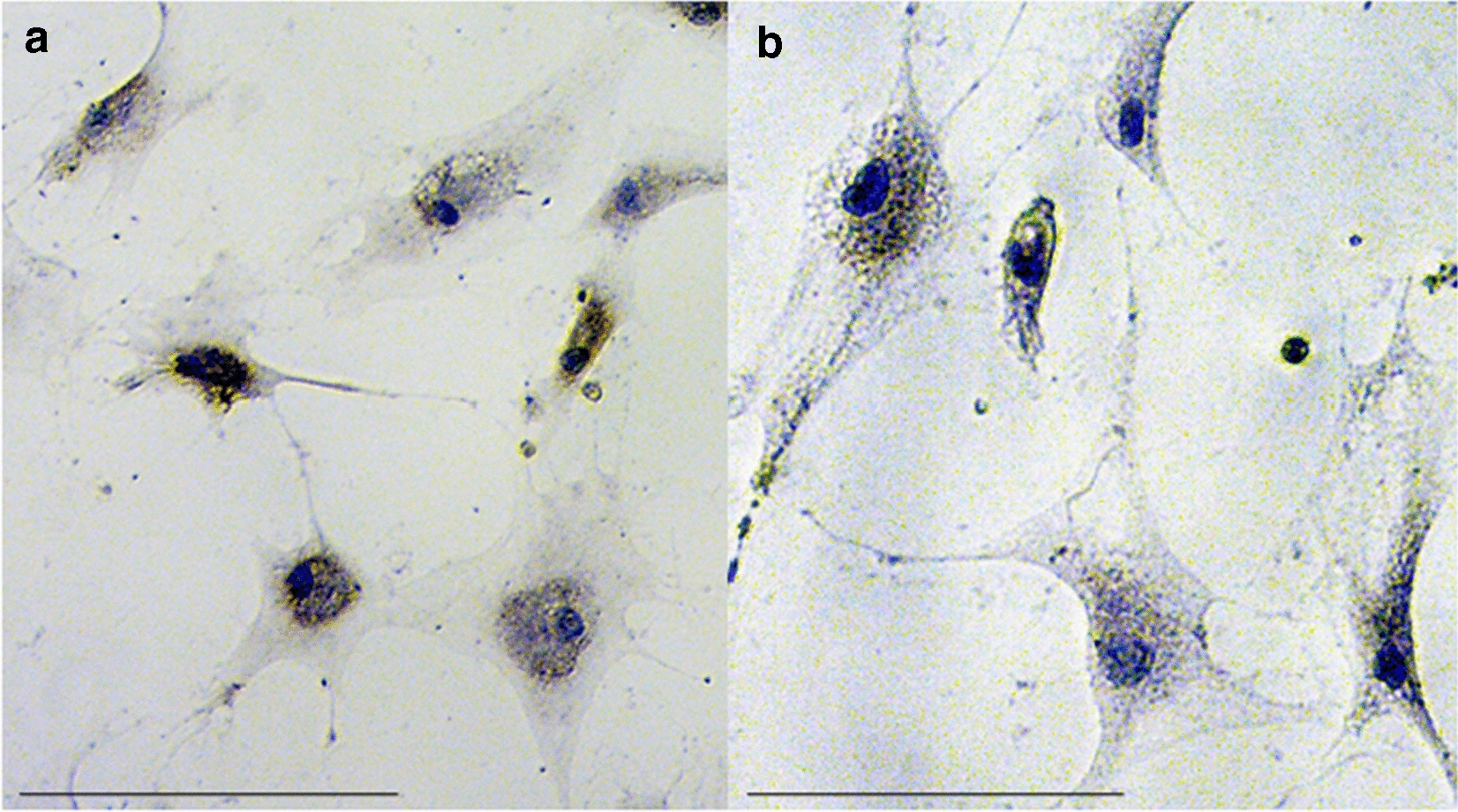
TRAP staining of PPFs (**a**) and SFs (**b**) cultivated in monolayer culture. No TRAP-positive cells are seen. The cells show a fibroblast-like, fusiform shape with a blue stained nucleus. Scale bar = 100 µm

All monolayer cultures were continuously screened histomorphologically and a spindle-shaped morphology of cells was observed. SFs, PPFs, and DFs did not stain positive for TRAP in monolayer cultures during the whole culture period (Fig. 1).

**Fig. 2:**
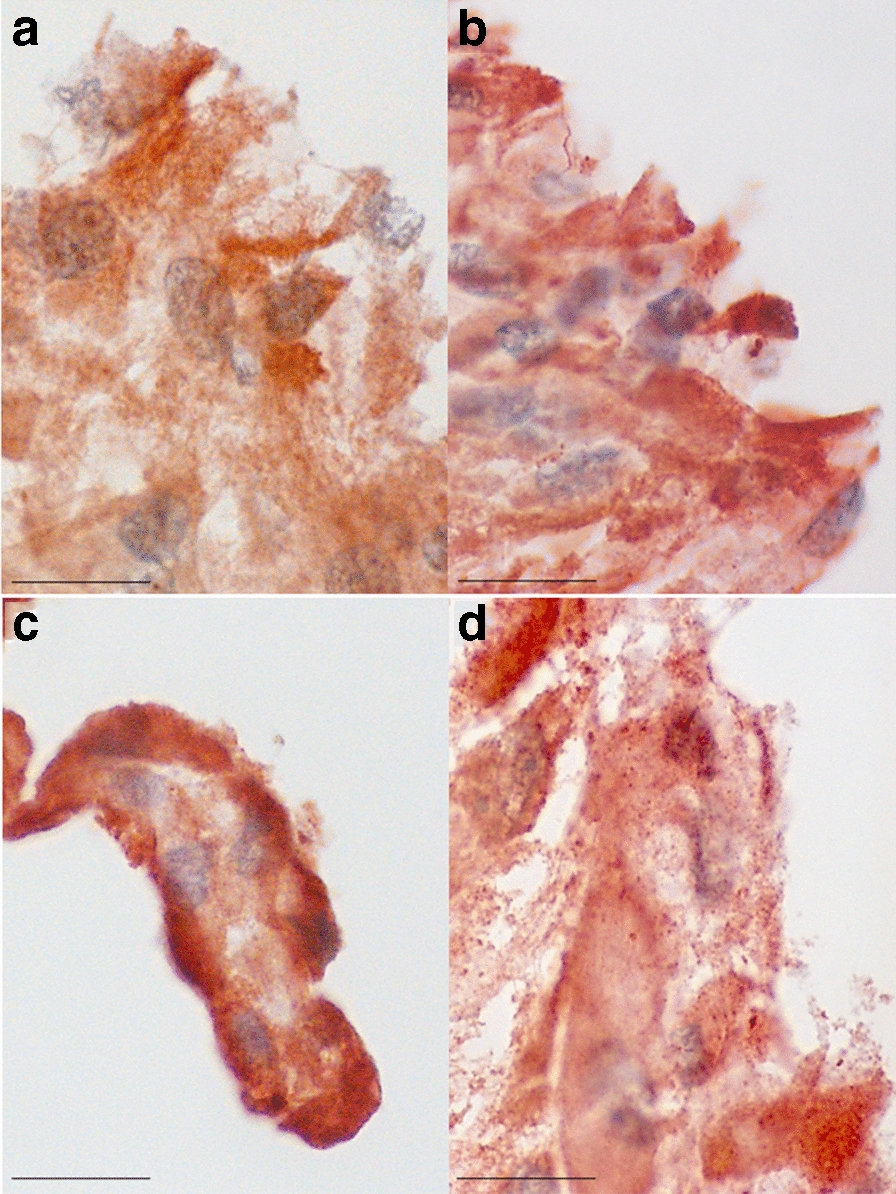
Immunostaining with the fibroblast marker S100A4 of multilayer cultures of PPFs (**a** and **b**) and SFs (**c** and **d**) on day 10 (**a** and **c**) and 21 (**b** and **d**). Scare bar = 20 µm

In transwell cultures, all cells stained positive for the S100A4 marker after 10 and 21 days (Fig. 2).

### Molecular analysis

**Fig. 3:**
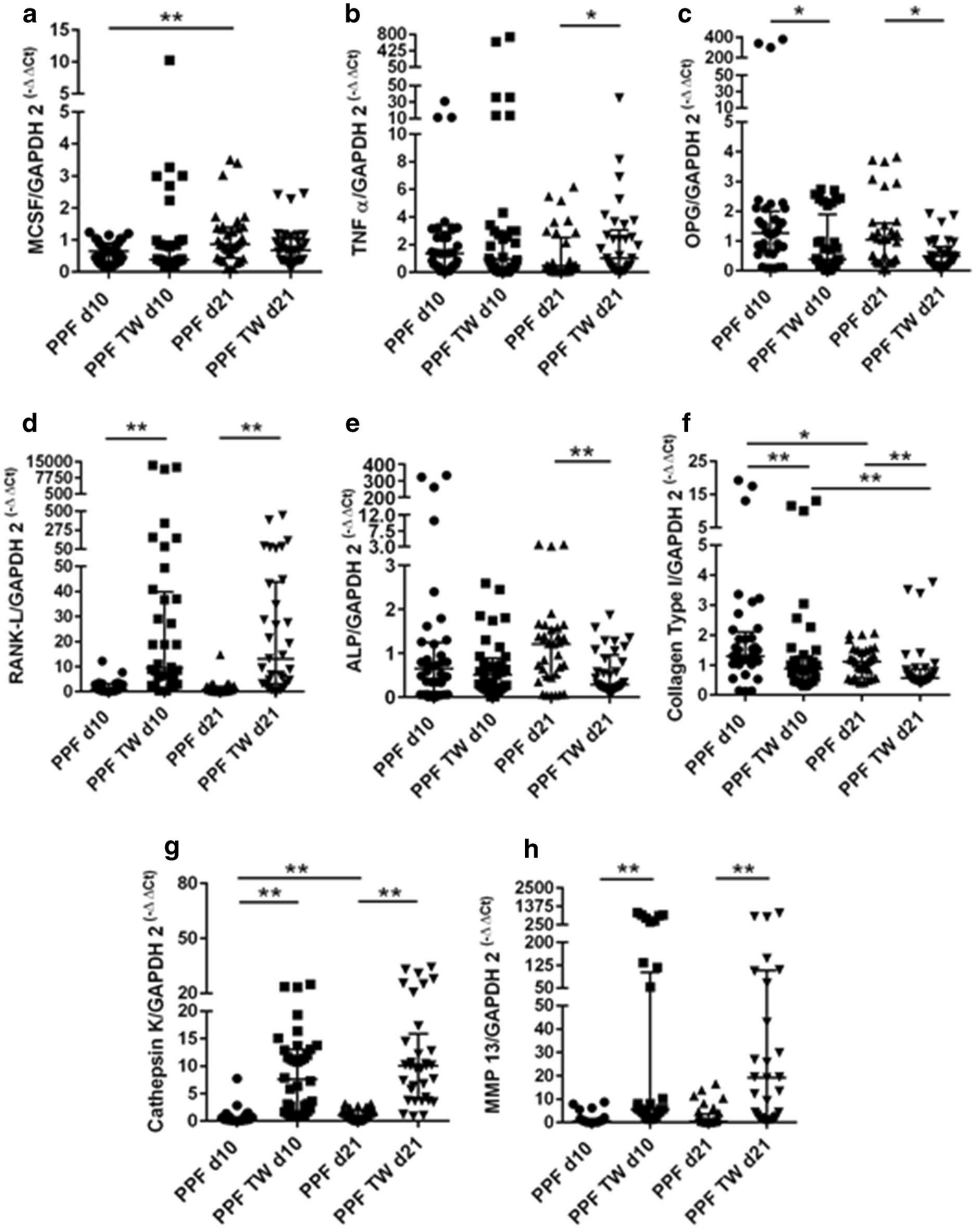
Relative mRNA expression of M-CSF (**a**), TNF-α (**b**), OPG (**c**), RANKL (**d**), ALP (**e**), COL1A1 (**f**), TRAP (**g**), cathepsin K (**h**), and MMP-13 (**i**) in PPF monolayer culture (PPF) and PPF transwell culture (PPF TW) on day 10 (d10) and day 21 (d21). Horizontal bars represent group medians and error bars IQR. *p* values are indicated with * = <0.05 and ** = <0.01

PPFs in transwell cultures showed significantly higher expressions for RANKL (both timepoints *p* < 0.001), cathepsin K (both *p* < 0.001), MMP-13 (both *p* < 0.001) on d10 and d21 (Fig. 3d, g, and h), and for TNF-α (*p* = 0.0445) on d21 (Fig. 3b) in comparison to monolayer cultures. For OPG (d10 *p* = 0.0281, d21 *p* = 0.0162), and COL1A1 (d10 *p* = 0.0034, d21 *p* = 0.0036), significantly lower expressions were observed in transwell cultures compared to monolayer cultures on d10 and d21 (Fig. 3c and f). For ALP (*p* = 0.0096), a significantly lower expression in transwell cultures was observed on d21 (Fig. 3e). No significant change of expression level for M-CSF of PPFs in transwell cultures compared to monolayer culture was noted (Fig. 3a).

**Fig. 4:**
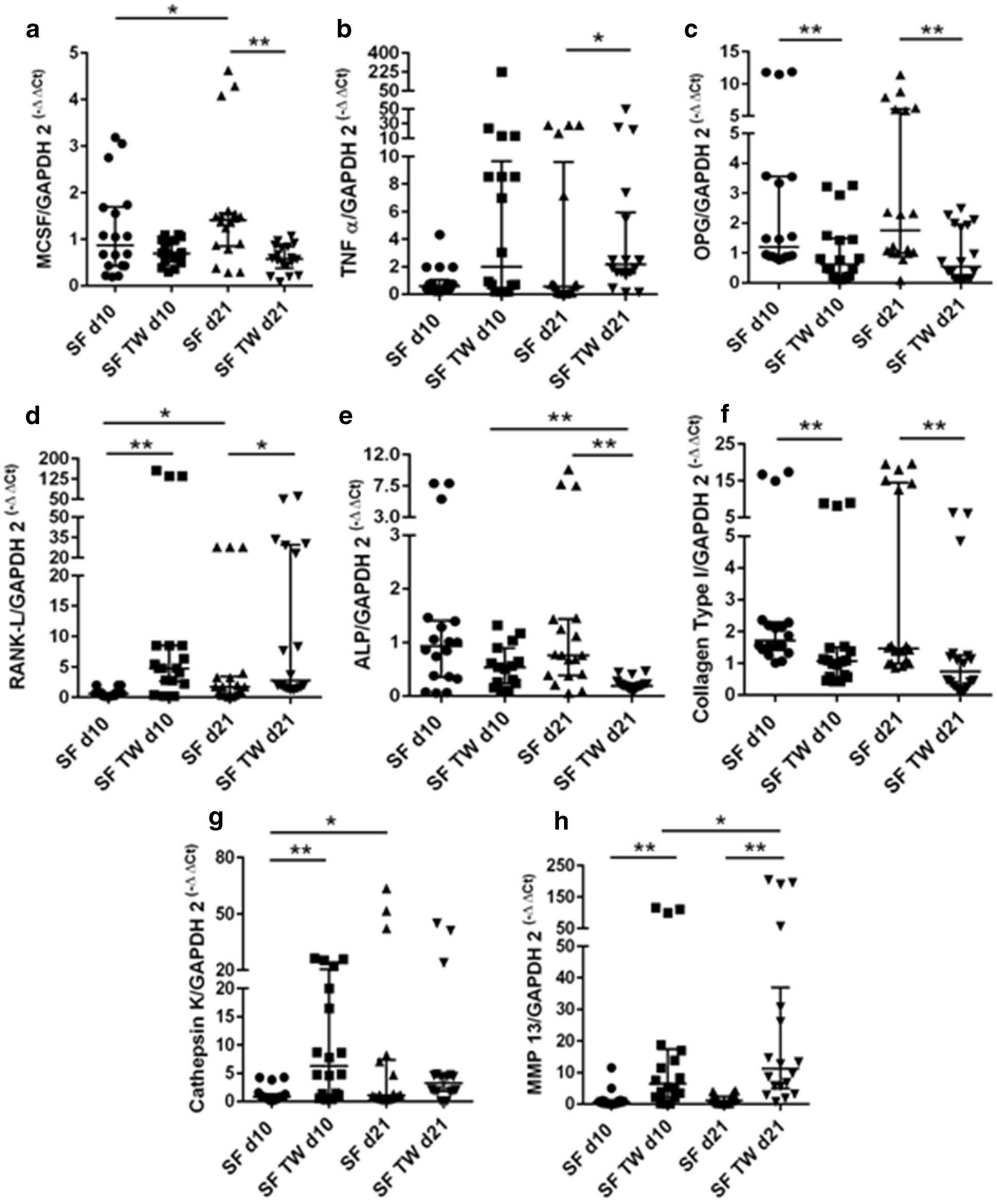
Relative mRNA expression of M-CSF (**a**), TNF-α (**b**), OPG (**c**), RANKL (**d**), ALP (**e**), COL1A1 (**f**), TRAP (**g**), cathepsin K (**h**), and MMP-13 (**i**) SF monolayer culture (SF) and SF transwell culture (SF TW) on day 10 (d10) and day 21 (d21). Horizontal bars represent group medians and error bars IQR. *p* values are indicated with * = <0.05 and ** = <0.01

SFs expressed significantly higher amounts of RANKL (d10 *p* < 0.001, d21 *p* = 0.0306) and MMP-13 (d10 *p* = 0.0012, d21 *p* < 0.001) in transwell cultures on d10 and d21 (Fig. 4d and h), of cathepsin K (*p* = 0.0004) on d10 (Fig. 4g) and of TNF-α (*p* = 0.0458) on d21 (Fig. 4b) compared to monolayer cultures. The expression of OPG (d10 *p* = 0.0029, d21 *p* = 0.003) and COL1A1 (d10 *p* = 0.0018, d21 *p* = 0.0033) was found to be significantly lower in SF transwell cultures than in monolayer cultures on d10 and d21 (Fig. 4c and f) and for M-CSF (*p* = 0.0004) and ALP (*p* = 0.0002) on d21 (Fig. 4a and e).

The main difference of expression changes between PPF and SF cultures was the lack of increased expression of cathepsin K (d21) and the decrease of M-CSF (d21) from monolayer to transwell culture in SFs.

When comparing expression levels over time, PPFs showed a significant decrease of COL1A1 (monolayer *p* = 0.0279, transwell *p* = 0.0152) expression from d10 to d21 (Fig. 3f) in both monolayer and transwell cultures and a significant increase of MCS-F (*p* < 0.001) and cathepsin K (*p* < 0.001) in monolayer cultures (Fig. 3a and g). SFs presented a significant increase of M-CSF (*p* = 0.0304), RANKL (*p* = 0.0101), and cathepsin K (*p* = 0.0208) (Fig. 4a, d and g) in monolayer cultures. In transwell cultures, SFs show a significant increase of ALP (*p* = 0.0026) and decrease of MMP-13 (*p* = 0.0304) (Fig. 4e and h) from d10 to d21.

**Fig. 5:**
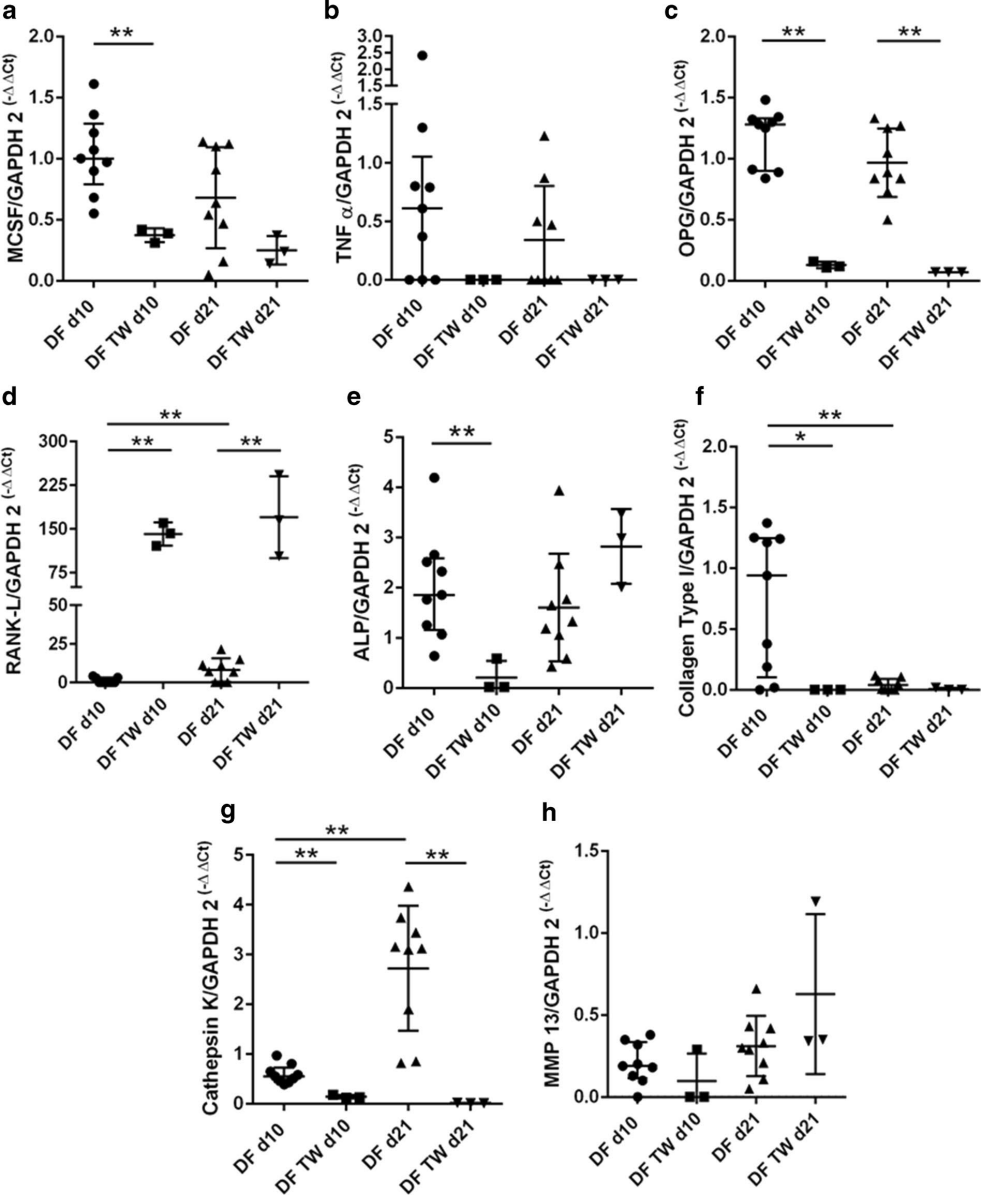
Relative mRNA expression of M-CSF (**a**), TNF-α (**b**), OPG (**c**), RANKL (**d**), ALP (**e**), COL1A1 (**f**), cathepsin K (**g**), and MMP-13 (**h**) in DF monolayer culture (DF) and DF transwell culture (DF TW) on day 10 (d10) and day 21 (d21). Horizontal bars represent group medians and error bars IQR. *p* values are indicated with * = <0.05 and ** = <0.01

DFs showed significant lower expression levels of M-CSF (*p* = 0.0091), ALP (*p* = 0.0091), and COL1A1 (*p* = 0.0273) on day 10 and of OPG (both days *p* = 0.0091) and cathepsin K (both days *p* = 0.0091) on days 10 and 21 in multilayer compared to monolayer cultures (Fig. 5a, c, e–g). The gene expression of RANKL (d10 *p* = 0.0045, d21 *p* = 0.0091) was significantly upregulated in DF multilayer cultures on d10 and d21 (Fig. 5d). Over time the expression values of RANKL (*p* = 0.0035) and cathepsin K (*p* = 0.0002) significantly increased, while the expression of COL1A1 (*p* = 0.0073) decreased in monolayer cultures for DFs (Fig. 5d, g and f).

**Fig. 6:**
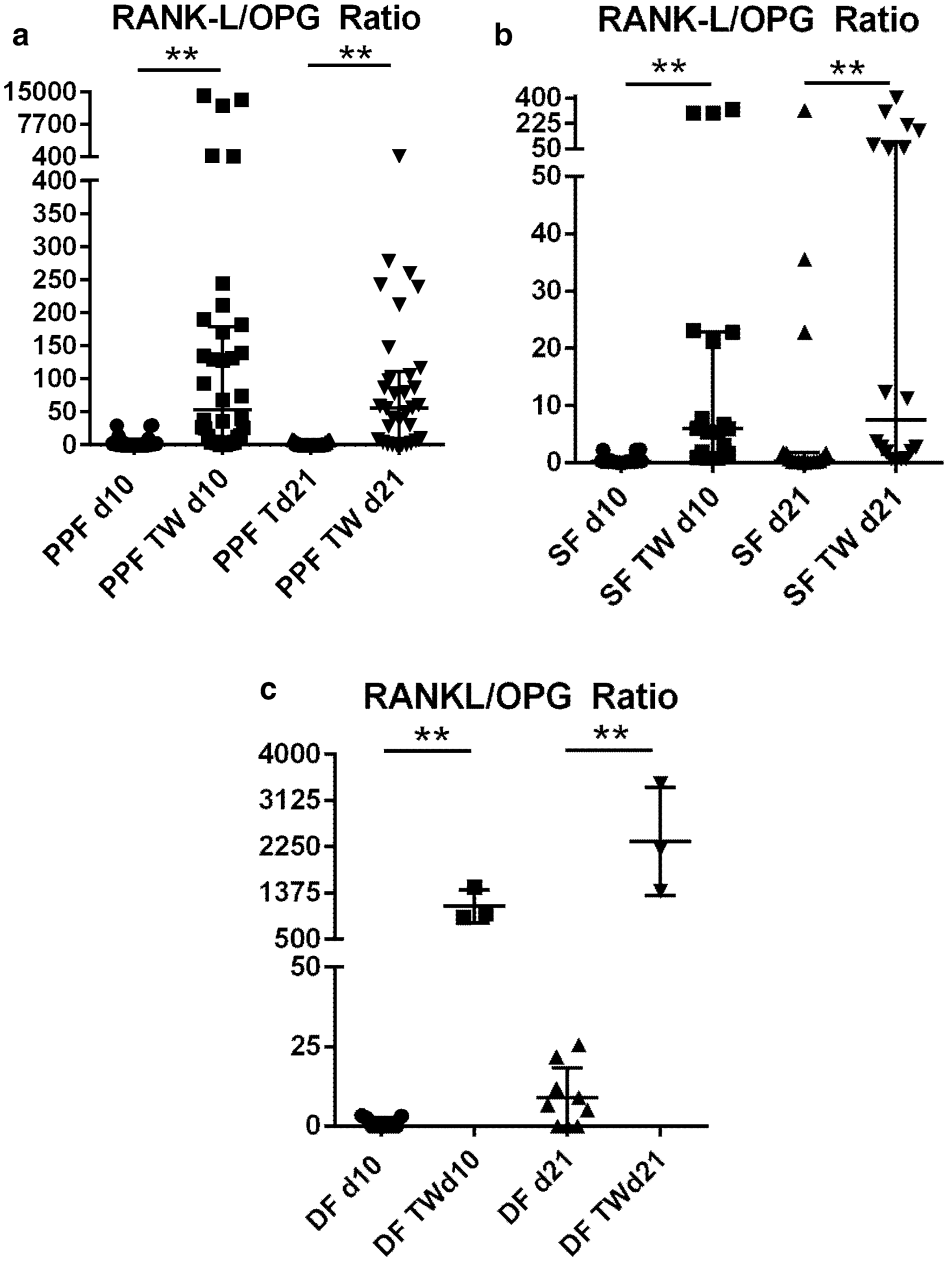
RANKL/OPG ratios of PPF, SF, and DF are presented. **a** PPF monolayer culture (PPF) and PPF transwell culture (PPF TW) on day 10 (d10) and day 21 (d21). **b** SF monolayer culture (SF) and SF transwell culture (SF TW) on day 10 (d10) and day 21 (d21). **c** DF in DF monolayer culture (DF) and DF transwell culture (DF TW) on day 10 (d10) and day 21 (d21). *P* values are indicated ** = <0.01

Regarding RANKL/OPG Ratios, PPFs as well as SFs and DFs showed all a significant increase when cultured on Transwell membranes on days 10 (*p* < 0.0001, *p* < 0.0001, and *p* = 0.0091) and 21 (*p* < 0.0001, *p* = 0.0008 and *p* = 0.0091), resulting in a more pro-osteoclastogenic environment (Fig. 6a–c).

## Discussion

Fibroblast-like cells from periprosthetic and from synovial tissue and dermal fibroblasts significantly increased the expression of RANKL, and significantly decreased the expression of ALP, COL1A1, and OPG in multilayer cultures without the addition of titanium, hydroxyapatite, or polyethylene particles or cytokine stimulation.

Regarding literature, suitable multilayer models for fibroblast-like cells from periprosthetic tissue and synovial tissue are scanty. We, therefore, mostly compared our findings to results from monolayer cultures.

There is lots of evidence that the function of fibroblast-like cells is far beyond creating a supporting tissue. Expression of RANKL, the final effector of osteoclastogenesis and bone resorption, has been shown to be increased in periprosthetic tissues [25]. It has been demonstrated that prostaglandin E2 (PGE2) signaling upregulates RANKL expression in PPFs [26]. PPFs can respond directly to wear debris, possibly via phagocytosis of titanium particles [4], stimulating RANKL expression by upregulating cyclooxygenase-2 (COX-2) expression through an oxidative stress-induced, calpain-dependent NF-κB-pathway [6]. Within this study the expression of RANKL was significantly increased in transwell cultures of all fibroblast-like cells and dermal fibroblasts without any further stimulus. It may be assumed that fibroblast-like cells are able to autoregulate RANKL expression by other mechanisms in multilayer cultures.

Fibroblast-like cells also express OPG, the decoy receptor that binds RANKL. OPG has been described to be upregulated in PPFs after titanium particle treatment in monolayer cultures [4] or by stimulation with TNF-α [8]. In multilayer cultures, a reduced OPG expression has been demonstrated for PPFs, SFs, and DFs. As periprosthetic tissues mostly are present with constantly very low OPG expression levels [25, 27, 28], it is of interest that an OPG reduction is obtained without the addition of further stimuli besides transwell culturing. We assume that OPG reduction, which is a pathomechanism in aseptic loosening, might be triggered by altered differentiation of fibroblast-like cells in transwell cultures [15].

The significant reduction of OPG combined with an increase in RANKL expression, resulted in a significant increase of the RANKL/OPG ratio within PPFs as well as SFs and DFs in transwell cultures. It showed a more pro-osteoclastogenic environment in all transwell cultures, independent of cell type. These findings match our previous results, where periprosthetic tissue (from which PPFs were isolated) was analyzed [25]. The multilayer aggregates seem to present an interesting approach to analyze fibroblasts within a cell-culture system mimicking in vivo situations more closely.

Cathepsin K and MMP-13 are known to be able to degrade the bone matrix [29–31] and are expressed by fibroblast-like cells. Cathepsin K has been demonstrated to be upregulated in PPFs after 1α,25-(OH)_2_ vitamin D_3_ stimulation [8] and is produced by synovial fibroblasts in rheumatoid arthritis [32, 33]. MMP-13 has been described to be highly expressed in periprosthetic tissues [34] by fibroblast-like cells which are often located in close vicinity to osteoclasts [35]. Both proteinases were significantly elevated in transwell cultures of PPFs and SFs, but did not show any changes in DF cultures. The cell-specific upregulation is an indicator for a cell-type specific enzymatic activity within multilayer cultures, which renders unspecific effects due to solely increased cell density unlikely.

Stimulation with titanium particles has neither led to an upregulation of TNF-α in PPFs nor in SFs in monolayer cultures [4], whereas the addition of substance P [11] or hydroxyapatite particles [36] increased the TNF-α production in PPFs. TNF-α showed an upregulation of expression for PPFs and SFs in the transwell system on day 21. As TNF-α plays a prominent role in regulating RANKL and OPG production [4, 8], the increase of TNF-α within multilayer cultures of fibroblast-like cells is another finding, which makes a cell-type specific effect more likely.

Another cytokine, M-CSF, had shown an upregulation in PPFs due to stimulation with TNF-α or titanium particles 12 h after stimulation. The expression, however, re-decreased after 48 h [4]. M-CSF was hardly regulated by transwell culturing. The constant expression of M-CSF might not be displaying early events of M-CSF regulation and should, therefore, be re-examined in the early hours after treatment.

A general problem of studies working with fibroblast-like cells is to characterize the cells and exclude contamination with other cell types. A standard protocol was used within this study [10], and PPFs and SFs were characterized by positive staining for S100A4. To get a positive fibroblast control, we used standard DFs in mono- and multilayer cultures. Regarding the limitations of this study, a contamination with osteoblastic and osteoclastic cells cannot be excluded. The osteoblast marker ALP, which can also be expressed to a small extent by fibroblasts [37, 38], was found to be expressed by PPFs, SFs, and DFs. However, ALP expression stayed at very low levels or was downregulated in multilayer cultures. The downregulation of ALP combined with an upregulation of MMP-13, which is not expressed by osteoblasts [9], renders a strong contamination with osteoblastic cells unlikely. We cannot completely exclude a contamination within the PPF and SF cultures. This also applies to a contamination with osteoclasts and osteoclast precursors, but the TRAP staining was negative and, therefore, contamination is unlikely.

Transwell cultures have already been reported to improve differentiation of 3T3L1 immortalized fibroblasts [15]. Within this study, multilayer cultures of fibroblast-like cells and DFs showed a significant downregulation of the extracellular matrix protein, COL1A1. We assume that multilayer cultures specifically change differentiation potentials and lower the expression of supporting extracellular matrix proteins like COL1A1.

One factor to induce the change of differentiation might be the high cell density in the multilayer cultures which enforces intercellular connections and direct cell-to-cell contacts in the style of an organ culture on a substratum [39]. The pores of the transwell culture system provide more access for cells to the culture medium within this conglomerate. Another factor might be the composition and matrix elasticity of the substratum, which can play a role in developing differentiation properties of cells [16]. Focal adhesion might also be changed in transwell cultures and allow the fibroblast-like cells, depending on their origin, to develop invadopodia, which contain proteases [24]. Transwell cultures have also been described to induce hypoxic conditions [40]. Hypoxia in multilayer cultures, which has been discussed to be present in periprosthetic tissues [41], might be a further reason for the change of differentiation. Whether viability of cells is concerned has to be elucidated. Hypoxia might also be a trigger factor which stimulates the expression of MMPs and osteoclastogenic factors [12]. It is of major interest that multilayer cultures do neither need the addition of particle nor cytokine stimulation to substantially change the expression of markers in fibroblast-like cells.

## Conclusion

We conclude that transwell cultures significantly changed the expression of bone remodeling markers in PPFs, SFs, and DFs without any further stimulus. In transwell cultures, the expression of RANKL was significantly upregulated, whereas the expression of ALP, COL1A1, and OPG was significantly decreased in fibroblast-like cells and dermal fibroblasts. For cathepsin K, MMP-13 and TNF-α expression changes in transwell cultures were only observed for PPFs and SFs. Fibroblast-like cells and fibroblasts change differentiation when transferred from monolayer into a multilayer environment and this might play an essential role in understanding bone remodeling processes. The transwell system needs to be further examined as it might be a useful tool to mimic in vivo like results regarding molecular answers on implant applications by fibroblast-like cell cultures. The clinical and scientific aspect to gain further understanding of processes within periprosthetic tissues might be enhanced using multilayer transwell systems instead of monolayer cultures.

## Data Availability

The datasets during and/or analyzed during the current study are available from the corresponding author on reasonable request.

## Abbreviations

ABC: Avidin–biotin–peroxidase complex
ALP: Alkaline phosphatase
α-MEM: α-Minimal essential medium
COL1A1: Collagen-type 1A1
DFs: Dermal fibroblasts
FBS: Fetal bovine serum
GAPDH: Glyceraldehyde 3-phosphate dehydrogenase
IQR: Interquartile range
M-CSF: Macrophage colony-stimulating factor
MMP-13: Matrix metalloproteinase 13
OA: Osteoarthritis
OPG: Osteoprotegerin
PBS: Phosphate-buffered saline
PGE2: Prostaglandin E2
PPFs: Periprosthetic fibroblast-like cells
PPT: Periprosthetic tissues
RANKL: Receptor activator of nuclear factor (NF)-κB ligand
RT: Room temperature
SFs: Synovial fibroblast-like cells
ST: Synovial tissues
THA: Total hip arthroplasty
TKA: Total knee arthroplasty
TNF-α: Tumor necrosis factor alpha
TRAP: Tartrate-resistant acid phosphatase
3D: Three-dimensional

## References

[1] Beck RT, Illingworth KD, Saleh KJ (2012). Review of periprosthetic osteolysis in total joint arthroplasty: an emphasis on host factors and future directions. J Orthop Res.

[2] Gallo J, Goodman SB, Konttinen YT, Raska M (2013). Particle disease: biologic mechanisms of periprosthetic osteolysis in total hip arthroplasty. Innate Immun..

[3] Gallo J, Goodman SB, Konttinen YT, Wimmer MA, Holinka M (2013). Osteolysis around total knee arthroplasty: a review of pathogenetic mechanisms. Acta Biomater.

[4] Koreny T, Tunyogi-Csapo M, Gal I, Vermes C, Jacobs JJ, Glant TT (2006). The role of fibroblasts and fibroblast-derived factors in periprosthetic osteolysis. Arthr Rheum.

[5] Yao J, Glant TT, Lark MW, Mikecz K, Jacobs JJ, Hutchinson NI (1995). The potential role of fibroblasts in periprosthetic osteolysis: fibroblast response to titanium particles. J Bone Miner Res.

[6] Wei X, Zhang X, Flick LM, Drissi H, Schwarz EM, O’Keefe RJ (2009). Titanium particles stimulate COX-2 expression in synovial fibroblasts through an oxidative stress-induced, calpain-dependent, NF-kappaB pathway. Am J Physiol Cell Physiol.

[7] Pap T, Claus A, Ohtsu S, Hummel KM, Schwartz P, Drynda S (2003). Osteoclast-independent bone resorption by fibroblast-like cells. Arthr Res Ther..

[8] Mandelin J, Li TF, Hukkanen M, Liljestrom M, Salo J, Santavirta S (2005). Interface tissue fibroblasts from loose total hip replacement prosthesis produce receptor activator of nuclear factor-kappaB ligand, osteoprotegerin, and cathepsin K. J Rheumatol..

[9] Wagner S, Gollwitzer H, Wernicke D, Langer R, Siebenrock KA, Hofstetter W (2008). Interface membrane fibroblasts around aseptically loosened endoprostheses express MMP-13. J Orthop Res.

[10] Sabokbar A, Itonaga I, Sun SG, Kudo O, Athanasou NA (2005). Arthroplasty membrane-derived fibroblasts directly induce osteoclast formation and osteolysis in aseptic loosening. J Orthop Res..

[11] Qian YB, Zeng BF, Zhang XL, Jiang Y (2008). Substance P stimulates production of interleukin 1 beta and tumor necrosis factor alpha in fibroblasts from hip periprosthetic membrane. J Arthroplasty.

[12] Muller-Ladner U, Ospelt C, Gay S, Distler O, Pap T (2007). Cells of the synovium in rheumatoid arthritis. Synovial fibroblasts. Arthr Res Ther..

[13] Niedermeier M, Pap T, Korb A (2010). Therapeutic opportunities in fibroblasts in inflammatory arthritis. Best Pract Res Clin Rheumatol.

[14] Majety M, Pradel LP, Gies M, Ries CH (2015). Fibroblasts influence survival and therapeutic response in a 3D Co-culture model. PLoS ONE.

[15] Sabater D, Fernandez-Lopez JA, Remesar X, Alemany M (2013). The use of Transwells (TM) improves the rates of differentiation and growth of cultured 3T3L1 cells. Anal Bioanal Chem.

[16] Engler AJ, Sen S, Sweeney HL, Discher DE (2006). Matrix elasticity directs stem cell lineage specification. Cell.

[17] Koehler MI, Hartmann ES, Schluessel S, Beck F, Redeker JI, Schmitt B (2019). Impact of periprosthetic fibroblast-like cells on osteoclastogenesis in co-culture with peripheral blood mononuclear cells varies depending on culture system. Int J Mol Sci.

[18] Wedemeyer C, Kauther MD, Hanenkamp S, Nuckel H, Bau M, Siffert W (2009). BCL2-938C > A and CALCA-1786T > C polymorphisms in aseptic loosened total hip arthroplasty. Eur J Med Res..

[19] Bloemen V, Schoenmaker T, de Vries TJ, Everts V (2010). Direct cell-cell contact between periodontal ligament fibroblasts and osteoclast precursors synergistically increases the expression of genes related to osteoclastogenesis. J Cell Physiol.

[20] Granchi D, Amato I, Battistelli L, Ciapetti G, Pagani S, Avnet S (2005). Molecular basis of osteoclastogenesis induced by osteoblasts exposed to wear particles. Biomaterials.

[21] Hong H, Park YK, Choi MS, Ryu NH, Song DK, Suh SI (2009). Differential down-regulation of COX-2 and MMP-13 in human skin fibroblasts by glucosamine-hydrochloride. J Dermatol Sci.

[22] Cordonnier T, Layrolle P, Gaillard J, Langonne A, Sensebe L, Rosset P (2010). 3D environment on human mesenchymal stem cells differentiation for bone tissue engineering. J Mater Sci.

[23] Vandesompele J, DePreter K, Pattyn F, Poppe B, Van Roy N, De Paepe A (2002). Accurate normalization of real-time quantitative RT-PCR data by geometric averaging of multiple internal control genes. Genome Biol.

[24] Waldele S, Koers-Wunrau C, Beckmann D, Korb-Pap A, Wehmeyer C, Pap T (2015). Deficiency of fibroblast activation protein alpha ameliorates cartilage destruction in inflammatory destructive arthritis. Arthr Res Ther..

[25] Hartmann ES, Kohler MI, Huber F, Redeker JI, Schmitt B, Schmitt-Sody M (2016). Factors regulating bone remodelling processes in aseptic implant loosening. J Orthop Res..

[26] Tsutsumi R, Xie C, Wei X, Zhang M, Zhang X, Flick LM (2009). PGE2 signaling through the EP4 receptor on fibroblasts upregulates RANKL and stimulates osteolysis. J Bone Miner Res.

[27] Tomankova T, Kriegova E, Fillerova R, Luzna P, Ehrmann J, Gallo J (2014). Comparison of periprosthetic tissues in knee and hip joints: differential expression of CCL3 and DC-STAMP in total knee and hip arthroplasty and similar cytokine profiles in primary knee and hip osteoarthritis. Osteoarthr Cartil..

[28] Koulouvaris P, Ly K, Ivashkiv LB, Bostrom MP, Nestor BJ, Sculco TP (2008). Expression profiling reveals alternative macrophage activation and impaired osteogenesis in periprosthetic osteolysis. J Orthop Res..

[29] Ma GF, Ali A, Verzijl N, Hanemaaijer R, TeKoppele J, Konttinen YT (2006). Increased collagen degradation around loosened total hip replacement implants. Arthr Rheum.

[30] Everts V, Delaisse JM, Korper W, Niehof A, Vaes G, Beertsen W (1992). Degradation of collagen in the bone-resorbing compartment underlying the osteoclast involves both cysteine-proteinases and matrix metalloproteinases. J Cell Physiol.

[31] Everts V, Delaisse JM, Korper W, Beertsen W (1998). Cysteine proteinases and matrix metalloproteinases play distinct roles in the subosteoclastic resorption zone. J Bone Miner Res.

[32] Hou WS, Li Z, Gordon RE, Chan K, Klein MJ, Levy R (2001). Cathepsin k is a critical protease in synovial fibroblast-mediated collagen degradation. Am J Pathol..

[33] Hou WS, Li W, Keyszer G, Weber E, Levy R, Klein MJ (2002). Comparison of cathepsins K and S expression within the rheumatoid and osteoarthritic synovium. Arthr Rheum.

[34] Takei I, Takagi M, Santavirta S, Ida H, Ishii M, Ogino T (2000). Messenger ribonucleic acid expression of 16 matrix metalloproteinases in bone-implant interface tissues of loose artificial hip joints. J Biomed Mater Res.

[35] Delaisse JM, Andersen TL, Engsig MT, Henriksen K, Troen T, Blavier L (2003). Matrix metalloproteinases (MMP) and cathepsin K contribute differently to osteoclastic activities. Microsc Res Tech.

[36] Ninomiya JT, Struve JA, Stelloh CT, Toth JM, Crosby KE (2001). Effects of hydroxyapatite particulate debris on the production of cytokines and proteases in human fibroblasts. J Orthop Res..

[37] Costa-Rodrigues J, Teixeira CA, Sampaio P, Fernandes MH (2010). Characterisation of the osteoclastogenic potential of human osteoblastic and fibroblastic conditioned media. J Cell Biochem.

[38] Weiss MJ, Ray K, Fallon MD, Whyte MP, Fedde KN, Lafferty MA (1989). Analysis of liver/bone/kidney alkaline phosphatase mRNA, DNA, and enzymatic activity in cultured skin fibroblasts from 14 unrelated patients with severe hypophosphatasia. Am J Hum Genet.

[39] Trowell OA (1954). A modified technique for organ culture in vitro. Exp Cell Res.

[40] Murdoch AD, Grady LM, Ablett MP, Katopodi T, Meadows RS, Hardingham TE (2007). Chondrogenic differentiation of human bone marrow stem cells in transwell cultures: generation of scaffold-free cartilage. Stem cells..

[41] Madathil BK, Lin Q, Hew CL, Mohanty M (2010). Hypoxia-like effect of cobalt chromium alloy micro particles on fibroblasts in vitro. J Orthop Res.

